# Early transcriptomic responses of rice leaves to herbivory by *Spodoptera frugiperda*

**DOI:** 10.1038/s41598-024-53348-x

**Published:** 2024-02-03

**Authors:** Laëtitia Leclerc, Trang Hieu Nguyen, Pénélope Duval, Victoria Mariotti, Anne-Sophie Petitot, Julie Orjuela, Jean-Claude Ogier, Sophie Gaudriault, Antony Champion, Nicolas Nègre

**Affiliations:** 1 DGIMI, Univ Montpellier, INRAE, Montpellier, France; 2 DIADE, Univ Montpellier, IRD, Montpellier, France

**Keywords:** Plant stress responses, Microbiome, Gene expression

## Abstract

During herbivory, chewing insects deposit complex oral secretions (OS) onto the plant wound. Understanding how plants respond to the different cues of herbivory remains an active area of research. In this study, we used an herbivory-mimick experiment to investigate the early transcriptional response of rice plants leaves to wounding, OS, and OS microbiota from *Spodoptera frugiperda* larvae. Wounding induced a massive early response associated to hormones such as jasmonates. This response switched drastically upon OS treatment indicating the activation of OS specific pathways. When comparing native and dysbiotic OS treatments, we observed few gene regulation. This suggests that in addition to wounding the early response in rice is mainly driven by the insect compounds of the OS rather than microbial. However, microbiota affected genes encoding key phytohormone synthesis enzymes, suggesting an additional modulation of plant response by OS microbiota.

## Introduction

The molecular dialog between insect pests and their host plant is determinant in the issue of the interaction and therefore the damages generated in crop fields^1^. Chewing insects are recognized by two cues: the damage of plant tissue by the mastication process and the deposition on the wound of oral secretions (OS)^2^. OS are composed of saliva from the labial and mandibular glands and microbiota from the anterior part of the gut, as well as regurgitated food bolus^3^. Consequently, plants are faced with a mix of molecular signals of different origins. Damage-associated molecular patterns (DAMPs) are produced by the plant itself upon wounding. Oligosaccharides derived from plant damaged cell walls are one of the most typical representative of DAMPs elicitors^4^. Herbivore-associated molecular patterns (HAMPs) are molecules produced in the insect saliva and regurgitated in the OS^2,5,6^. HAMPs such as *ß*-glucosidase, fatty-acid-amino acid conjugates (FACs) and inceptins induce the plant defense response through their recognition by cell-membrane bound receptors such as pathogen recognition receptors (PRR) that then mediate the so-called pathogen-triggered immunity (PTI) of the plant^7^. Other molecules contained in the OS, termed effectors, can either suppress this response or induce other pathways of immunity (ETI: effector triggered immunity) through their recognition by intracellular receptors^2,5,6,8,9^. Microbiota present in the OS can also modulate plant defense response. For example, studies on *Coleoptera*/*Solanaceae* and *Lepidoptera*/*Brassicaceae* or *Solanaceae* or *Poaceae* interactions showed that OS microbiota modulates phytohormone-dependent plant defense^10–15^. The effect varies depending on the insect-host plant model and the bacteria species present in the OS and it is mediated by microbe-associated molecular patterns (MAMPs). How plant cells can integrate the various herbivory signals at the molecular level and mount an effective response to deter herbivores is currently an active area of research. While most of our knowledge on plant immunity is based on models such as *Arabidopsis thaliana* (Heynh, 1842), in-depth molecular analyses on models of agro-economic relevance also need to be conducted.

Rice (*Oryza sativa*, Linné, 1753) is the first cereal in the human diet in the world and rice fields are faced with many insect pests such as planthoppers and leafhoppers (https://www.fao.org/3/y6159t/y6159t02.htm). While many mechanisms of rice immunity against microbial pathogens have been described, rice defense against herbivorous insects is less known. The PRR *OsLRR-RLK1*, a gene encoding a leucine-rich repeat receptor-like kinase involved in the perception of HAMPs, has been described as responding to the fall armyworm (FAW, *Spodoptera frugiperda*, J.E Smith, 1797) OS but not to mechanical wounding^16^. This perception initiates an early signaling cascade involving the mitogen-activated protein kinase cascade (MAPK) pathway, increased cytosolic Ca^2+^ and a production of Reactive Oxygen Species (ROS)^1^. During an attack by the rice striped stemborer (SSB, *Chilo suppressalis*, Walker, 1863), *OsMAPK3* positively regulates defense responses by modulating jasmonates (JA) biosynthesis^17^. The transcription factor (TF) *OsWRKY70* is regulated by *OsMPK3* and *OsMPK6*, and induces an increased level of JA, ethylene (ET) and trypsin proteinase inhibitor activity^18^.

The early signaling cascade induces the production of phytohormones involved in defense. For exemple, JA biosynthesis is rapidly triggered after attack, resulting in a JA burst^19^. This JA burst is mainly induced by the wounding component of herbivory^19–21^. Rice can also recognize elicitors from the OS of various herbivores such as *Spodoptera mauritia* (Boisduval, 1833), *Mythimna loreyi* (Duponchel, 1827), and *Parnara guttata* (Bremer & Grey, 1852) and, in response, accumulates higher levels of JA/JA-Ile (jasmonate-isoleucin) and/or defense related secondary metabolites compared with wounding alone^22,23^. SSB larvae also increase JA levels in rice^24^ and induce the expression of *OsLOX9* and *OsAOS2*, two genes encoding enzymes involved in the biosynthesis of JA^25^. Salicylic acid (SA) is also induced by herbivory. Upon SSB attack, the *OsPAL1* and *OsPR-1a* genes, involved in the SA signaling pathway, are induced 24 h after treatment^25^. ET is also induced in rice by SSB and *Mythimna separata* (Walker, 1865)^26^.

These signal molecules trigger defense responses by producing secondary metabolites. These metabolites can be either toxic compounds involved in direct insecticidal defense^27,28^ such as phytoalexins that include flavonoids or volatile secondary metabolites that attract natural predators of the insect such as parasitoids^29,30^ and are grouped into three main categories: green leaf volatiles (GLVs), aromatic compounds and terpenes. To counter these defenses, herbivorous insects developed many strategies including suppression of Herbivore-Induced Volatile (HIPV) induction or detoxification of secondary plant metabolites. For example, glucose oxidase produced in the saliva of *Helicoverpa zea* (Boddie, 1850) is able to counteracts the production of nicotine, a toxic alkaloid in tobacco (*Nicotiana tabacum*, Linné, 1753), induced by the caterpillar feeding^31^. These papers have described the induction of JA by wounding and of SA by HAMPs during herbivory. However, only five papers were carried out on the overall transcriptomic response of rice to herbivores, but without separating the different cues of herbivory^32–36^. Which molecular pathways are modulated by these cues remains to be discovered.

FAW, is an agricultural pest native of the Americas^37^. While its chewing herbivorous, polyphagous caterpillar has been reported on 353 plant species^38^, FAW causes significant damage to crops of economic interest and has a preference for *Poaceae*, in particular maize (*Zea mays*, Linné, 1753)^39,40^. During the last decade, it has dispersed around the world. It was detected for the first time in 2016 in Africa where it spread rapidly, in 2018 in Asia and in 2020 in Oceania^39,41^. International trade and global warming foster its access to new territories^42^, which poses a risk to global food security. While maize is generally described as the most favorable plant for FAW in both its native and invasive range^43^, rice, on the other hand, seems deleterious for FAW^44–46^, even though sporadic outbreaks are being regularly reported in rice fields in the Americas^47^ and is listed among the preferred host plants for FAW. This suggests that FAW may have evolved strategies to counter rice’s defense system against herbivorous insects.

Insects are associated with a cohort of microorganisms (bacteria, *Archaea*, fungi, protozoa and viruses) that make up its microbiota^48^. Insect-associated microorganisms increase nutrient utilization capacity, regulate insect immune responses, provide protection against parasites and predators^49–51^, and detoxify secondary metabolites produced by plants^52^. In FAW, the bacterial microbiota has been extensively documented^11,53–56^. While the role of bacteria in OS in modulating plants (corn and tomato, *Solanum lycopersicum*, Linné, 1753) defense response to herbivory has been recently characterized^10,11,13^, the molecular pathways by which microbiota interferes with plant immunity are yet to be described. Moreover, the bacterial microbiota of FAW OS has never been characterized by metagenomics.

The objective of this study was to decipher the early responses of rice to herbivory cues: mechanical wounding and OS. To this end, we have carried out a global transcriptomic analysis of rice response in a herbivory-mimic procedure using deposition of FAW OS on wounded leaves*.* RNA-seq performed on leaves samples collected two hours after the different treatments identified rice genes responding to specific cue or co-modulated. Notably, we show that JA-dependent response is mainly triggered by mechanical wounding but that deposition of insect OS triggers additional response specific of defenses against insects and is likely due to activation of SA pathway. In addition, we identify for the first time a list of genes (~ 2500) including 209 TFs, specifically responding to FAW OS. To understand the implication of oral bacterial microbiota, we first analyzed the bacterial composition of FAW using cultural and metabarcoding approaches and developed dysbiotic OS. Two hours after treatment, modification of the microbiota did not have a massive effect on early rice response although 33 genes with microbiota-attenuated expression levels were identified. The role of these microbiota specific genes in modulating overall plant defenses remains to be determined.

## Materials and methods

### Insect rearing and bioassays

FAW larvae (rice-strain)^49^ were reared on an artificial diet, the Poitout^57^, in DGIMI insect facility at 23 ± 1 °C, with a photoperiod of L16:D8 and relative humidity of 40 ± 5%. This laboratory strain has been cultivated from a Florida isolate since 2011 and corresponds to the genome assembly published in^40^. Experiments were performed in an oven at 23 ± 2 °C located in the experimental facility PIQ dedicated to quarantine insect species in accordance with the 2008/61/CE European directive. For gut treatment, fifth instar larvae were fed with Poitout supplemented with antibiotics (5 µg ampicillin and 20 µg erythromycin/g of Poitout) or equivalent volume of deionized water (control). After three days on this diet, the larvae reached sixth instar. They were surface disinfected with ethanol (70%), held with a flexible forceps, which stimulates larvae regurgitation. OS were recovered with a sterile Pasteur pipette, 2–3 µL/larvae. OS from 75 individual larvae treated in the same way were pooled and were named OS+ and OS− when larvae were fed with artificial diet or with artificial diet treated with antibiotic, respectively. OS were stored at − 20 °C for subsequent uses.

Midguts were harvested from larvae at the L6 medium stage using sterile forceps and scissors, then stored in a 1.5 mL Eppendorf tube containing 1 mL 1X phosphate buffered saline (PBS). A total of three midguts were collected and stored at − 20 °C prior to DNA extraction.

### Identification of cultivable bacteria contained in OS

Before freezing, dilutions of OS were plated on 1.5% nutrient agar medium (Difco) at 28 °C for 24 h. Two different morphotypes of colonies were observed. Genomic DNA of two representative isolated colonies was extracted using the QiAmp extraction kit (Qiagen) according to manufacturer recommendations. To identify bacteria, PCR amplifications of the 16S rRNA gene were performed with the following primers: 63Bis (F) 5′-GAAGAGTTTGATCATGGCTC-3′ and 153Rev (R): 5′-AAGGAGGTGATCCAGCCGCA-3′. Briefly, about 10 ng of DNA were used and amplified in a Bio-Rad thermocycler (Bio Rad, USA) with GoTaq G2 Flexi DNA Polymerase (Promega, USA) according to manufacturer recommendations. Amplification products were sequenced at MWG Eurofin center (Germany) and compared to NCBI database by BLAST (https://blast.ncbi.nlm.nih.gov/Blast.cgi).

### DNA extraction for bacterial metabarcoding

Whole DNA extraction was performed on pool of 75 individuals OS (volume of 50–100 µL) and on one midgut (volume of 200 µL of LB) from L6 stage of FAW larvae. Cells were first lyzed by being frozen at − 80 °C for 15 min, then heated at 80 °C for 20 min. 100 μL of Quick Extract lysis solution (Bacterial DNA extraction kit from Epi-centre, USA), 1 μL of Ready-Lyse Lysozyme Solution (Epi-centre, USA) and 20 µL EDTA (0.5 M, pH 8) were added. A mechanical lysis with six 2 mm-glass beads was preceded in 3 cycles of 7 m/s of 40 s by using the FastPrep apparatus (MP Biomedicals, Illkirch-Graffenstaden, France). Complete lysis of both insect and prokaryotic cells was obtained by incubation at room temperature for 90 min followed by an incubation at 55 °C with shaking at 17,000 g with 10 µL of Proteinase K (20 mg/mL) until the solution cleared. RNA was eliminated with 10 μL of RNaseA 20 mg/ml (Invitrogen PureLinkTM RNaseA, France) for 15 min at 37 °C. After a last incubation with 100 µL of Protein precipitation solution (Kit Wizard, Promega, France) for 5 min on ice, samples were centrifuged 5 min at 17,000 g at 4 °C and the supernatant was recovered. DNA contained in midgut samples were precipitated with isopropanol. Due to the presence of polysaccharides, OS samples were subjected to phenol chloroform and chloroform treatments. DNA contained in OS samples were precipitated with absolute ethanol and washed with 70% ethanol. Both extracted DNA were resuspended in 50 μl ultrapure water and stored at − 20 °C.

### Library preparation and sequencing

We used a two-stage PCR method to produce amplicon libraries for sequencing on an Illumina MiSeq. PCR were conducted in triplicates. Our protocol also included negative and positive controls composed of sterile water and *Enterococcus mundtii* genomic DNA, respectively. Briefly, the first PCR targets the hyper variable V3-V4 region (~ 460 bases) with the universal primers F343 and R784 containing a 5' nucleotide overhangs with adaptors for the indexation of library and was performed with the iProof TM DNA Polymerase (Bio-Rad) as previously described^58^. These extensions aimed at anchoring a second round of PCR that introduced indexes and completed Illumina adapters. After a magnetic bead purification (Clean PCR, Proteigene, France), the second round of PCR was performed in a total volume of 18 µL (5 µL of first round PCR products, 9 µL Phusion® High-Fidelity PCR Master Mix, NEB, France, 2 µL I5 index-adapter, 2 µL I7 index-adapter). Cycling conditions were as follows: 95 °C for 3 min then 10 cycles of 95 °C 30 s., 55 °C 30 s., 72 °C 30 s. then final elongation of 5 min. at 72 °C. A set of 384 index pairs based on the unique dual (UD) index set from IDT was used to tag all the samples and multiplex them on a single MiSeq run. After a purification with magnetic beads, these final PCR products were multiplexed and paired-end sequenced on a MiSeq Illumina sequencer using MiSeq Reagent Kit v3 (600-cycles; Illumina). All procedures from the second PCR to the Illimina sequencing have been completed at the Genseq platform (University of Montpellier, France).

### Sequence data processing and taxonomic assignation

Sequencing reads obtained were processed according to the FROGs pipeline^59^. Pre-processing analyses were performed as previously described^60^. Sequences were assigned with RDP Classifier^61^ and the 16S rRNA database Silva^62^.

### Bacterial community and statistical analyses

Operational taxonomic unit (OTU) diversity and statistical analyses were carried out with the R packages Phyloseq, Vegan and Ampvis2 as previously described^60^.

### Plants and growth conditions

*Oryza sativa*, japonica cv Kitaake wild-type (WT) rice seeds were used for the experiments. Seeds were obtained from Pamela C. Ronald laboratory (U.C. Davis, U.S.A.) which originally sequenced its genome. Since the Kitaake cultivar is registered on the *International Treaty on Plant Genetic Resources for Food and Agriculture*: *ITPGRFA* all related methods were performed in accordance with the relevant guidelines and regulations. Seed coats were removed, and seeds were sterilized in 70% ethanol for 1 min, then in 3.7% bleach for 30 min. They were rinsed six times with sterile water and sown on half‐strength Murashige & Skoog (^½^MS) medium including Gamborg B5 vitamins (Duchefa Biochemie BV, Haarlem, the Netherlands) supplied with 0.5% plant agar (Duchefa), pH 5.8. After two days in a culture chamber (27 °C, 70% relative humidity, 12 h photoperiod, 12,000 lx light intensity), germinated seeds were vertically placed in a square Petri plate contain ^½^MS + B5 medium supplied with 0.7% plant agar in its one third inferior part. Five seeds were placed per plate and grown for four days under the same conditions.

### Wounding and deposition of OS on wounded rice leaves

Different treatments were performed to mimic FAW attack on rice leaves with a simulated herbivory system: (i) control treatment (**C**) where leaves were not wounded, (ii) mechanical wounding (**W**) where each leaf was wounded three times with a metal forceps and 1.5 µL of sterile water is deposited per wound, , (iii) mechanical wounding and OS (**WOS+**) treatment where 1.5 µL of OS is deposited per wound and (iv) mechanical wounding and dysbiotic OS (**WOS−**) treatment where 1.5 µL of dysbiotic OS is deposited per wound. After treatment, the plants were left for 2 h in the culture chamber. Then the leaves from five seedlings were pooled, and immediately frozen in liquid nitrogen before being stored at − 80 °C. Four biological replicates were performed.

### Total RNA extraction

Total RNA from rice leaves was extracted with the RNeasy Plant Mini kit (Qiagen) with an additional DNase treatment step to remove genomic DNA contamination. The concentration and purity of the RNA samples were measured with a NanoDrop spectrophotometer.

### RNA-seq data and bioinformatics analysis

cDNA library preparation and sequencing were performed on four replicates by Novogene (Novogene, Cambridge, UK) using the Illumina NovaSeq platform generating 150-bp paired-end reads. Raw data have been submitted to ArrayExpress with the accession number **E-MTAB-13227** (https://www.ebi.ac.uk/biostudies/arrayexpress/studies/E-MTAB-13227). Bioinformatics analyses were performed on the iTrop platform's computing cluster, certified EURO-QUALITY SYSTEM ISO 9001. The TOGGLe (TOolbox for Generic NGS anaLyses)^63^ workflow was used to perform quality analysis, cleaning, mapping, and to produce count tables. Briefly, the quality of fastq files was checked with the FASTQC software. For the cleaning step, the ATROPOS software was used to eliminate the first nine nucleotides and adapter sequences. Clean reads were then mapped with the Hisat2 and Stringtie softwares on the Nipponbare genomes (MSU and RAPDB) using an annotation file. The count tables were generated from the gtf files using an in-house script. Sequencing statistics of number of raw reads per sample, cleaned reads and mapped reads on rice transcriptome are presented in Supplementary Table 1.

For gene expression analyses, we used the R-shiny DIANE tool (Dashboard for the Inference and Analysis of Networks from Expression data)^64,65^. This online application allowed us to perform data normalization and differential expression analysis using edgeR^66^, with an absolute log_2_-fold change of 1 (or − 1) and a *p*-value of 0.01 (FDR: False Discovery Rate) as selection criteria of differentially expressed genes (DEGs), and gene ontology (GO) enrichment of the DEGs.

### Real-time quantitative PCR

The same biological replicates used for RNA-seq were analysed by quantitative RT-PCR. First-strand cDNAs were synthesized from 1 µg of total RNA in 20 µl final volume using an oligo-dT(18)-MN primer (Eurogentec, France) and the Omniscript RT kit (Qiagen). Quantitative-PCR assays were performed on cDNAs samples (diluted 1/50e) in an Mx30005P thermal cycler (Stratagene, USA) using the Brilliant III Ultra-fast SYBR® Green QPCR Master mix with low ROX (Agilent, Santa Clara, CA, USA) and specific primers (final concentration 200 nM; Supplementary Table 2). Amplification and melting curves were generated to verify the efficiency and specificity of the qPCR. Experiments were performed with four biological replicates and two technical replicates. Relative expression of genes were analyzed with the delta delta Ct method: [ΔΔCt = ΔCt(treated sample) − ΔCt(untreated sample)] where [ΔCt = Ct(gene of interest)  − Ct(reference gene)]^67^. Statistical analyses were performed using GraphPad Prism (version 9.3.0).

## Results and discussion

### Transcriptomic analysis of rice leaves deciphers early response to herbivory signals

We performed an herbivory-mimick procedure on rice leaves similar to previously established protocols^23^. The mechanical wounding condition (W) consisted of leaves that have been damaged with a forceps and a droplet of water deposited on the wounds. The WOS+ condition consisted of similarly wounded plants where OS from FAW have been deposited on the wounds instead of water. Control (C) consisted of untreated rice leaves. Transcriptomic experiments were conducted on RNA extracted from rice leaves collected 2 h after treatment. Sequencing and alignement statistics, as well as accession numbers, are presented in Supplementary Table 1.

**Figure 1:**
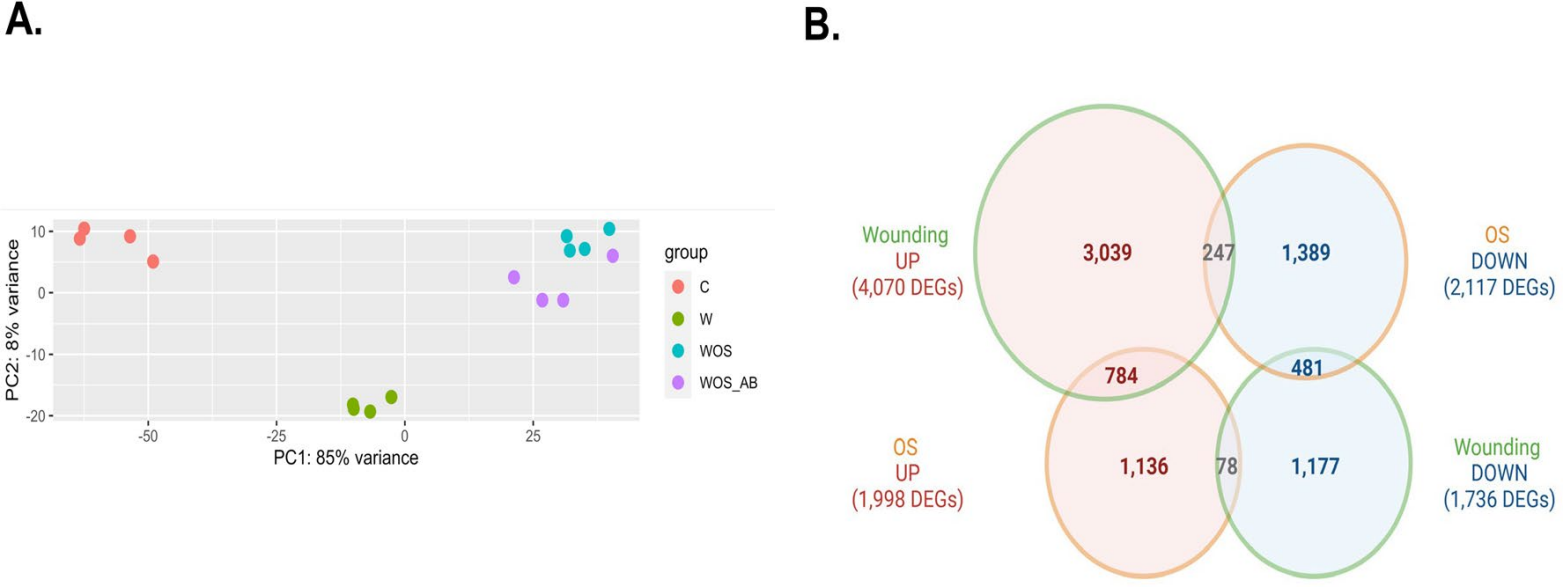
Global transcriptional response of rice genes to FAW herbivory-mimick treatments. (**A**) Principal component analysis (PCA) of normalized read counts showing, for each principal component, the part of global variability explained for each of the four replicates and the treatments: control (C), mechanical wounding (W), herbivory (WOS = WOS+) and herbivory with dysbiotic microbiota (WOS_AB = WOS−). (**B**) Venn diagram showing the number of over-expressed (UP) and under-expressed (DOWN) differential expressed genes (DEGs) in the wounding (W vs C) and oral secretion (WOS+ vs W) comparisons. The number of common DEGs in the four possible intersections between the two experiments is indicated.

A principal component analysis (PCA) on normalized read counts per gene shows that the four biological replicates for each condition are grouped together. Principal component 1 explained 86% of the variability, allowing to separate the different treatments (Fig. 1A). Principal component 2 explains 9% of the variability mainly present in the C group (Fig. 1A).

The global effect of herbivory can be obtained by comparing WOS+ versus C. We identified 9956 DEGs (5609 DEGs UP; 4347 DEGs DOWN) that could be modulated by either mechanical wounding or OS. To investigate rice responses to the different components of herbivory, we first measured the effect of mechanical wounding by the identification of DEGs between the W and C conditions. Our analysis identified 5806 DEGs, most of them over-expressed (UP: 4070) and only one third under-expressed (DOWN: 1736) (Fig. 1B, Supplementary Table 3). We then compared WOS^+^ versus W treatments to identify genes whose expression is influenced by the deposition of FAW OS. We observed a huge effect of OS application with the identification of 4115 DEGs, with a similar number of over-expressed (UP: 1998) compared to under-expressed ones (DOWN: 2117) (Fig. 1B, Supplementary Table 3). We intersected these two DEG lists and identified 4216 DEGs (3039 UP and 1177 DOWN) responding to damage only (D in the subsequent figures) and 2525 genes (1136 UP and 1389 DOWN) responding to the herbivore OS only (H in the subsequent figures). We found 1590 DEGs that were co-modulated by mechanical wounding and OS (DH in the subsequent figures) (Fig. 1B). To validate the RNA-Seq data and illustrate the different categories of differential expressions, we performed independent quantitative RT-PCR using three genes retrieved from the D list (*OsAOC, OsMYC2* and *OsJAZ8*), the H list (*OsJiOsPR10, OsLRR-RLK1* and *OsTPS31)* and the DH list (*OsJAZ9, OsJAZ11* and *OsTPS30*) (Supplementary Fig. 1A, B, C). When comparing the relative expression of those genes in qPCR with RNA-seq quantification, we could retrieve a similar expression profile.

The D UP list is enriched in 37 Gene Ontology (GO) terms (25 biological process (BP) terms, 9 cellular component (CC) terms and 3 molecular function (MF) terms) and the DOWN DEGs are enriched in 15 GO terms (7 BP, 1 CC, 7 MF) (Supplementary Table 4). Among the most significative BP GO enrichment of the UP DEGs are response to JA, response to SA, and response to oxidative stress. Among all regulations performed by JA, it is generally assumed that a specific branch of JA signaling responds to wounding alone^68,69^. While SA-dependent resistance to pathogens is thought to be induced mainly via the detection of HAMPs, it has been shown that it can also be mediated by the detection of DAMPs such as oligogalacturonides, systemin^70^ and esDNA (extracellular free DNA)^71^. The DAMPs usually originate from the degradation of the plant cell wall and stimulate the production of ROS^72^. We also observed a specific enrichment for wounding response, sugar metabolism and an enrichment in ribosomal synthesis that may reflect an overall metabolic response to cellular damages.

GO enrichment in the H list indicates that the most significative BP terms for the upregulated DEGs correspond to JA pathway genes, SA pathway genes, as well as functions associated to response to herbivory (Supplementary Table 4). Given that the same phytohormone pathways are found enriched in both wounding and OS responses, there is likely co-modulation of gene expression by the two stimuli. In the DH list (1590 DEGs), we distinguished four different categories depending on whether the response is synergistic (784 DEGs named ‘UP-UP’ and 481 DEGs named ‘DOWN-DOWN’) or antagonistic (247 DEGs named ‘DOWN-UP’ and 78 DEGs named ‘UP-DOWN’) (Fig. 1B). The 784 'UP-UP' DEGs are enriched in 28 GO terms mostly linked to JA-dependent response (Supplementary Table 4). No enrichment has been detected for the 481 'DOWN-DOWN' genes, as well as for the antagonistic DEGs, most likely due to their small number.

In summary, this analysis identified for the first time a set of rice genes whose expression in leaves is specifically affected a mere 2 h upon deposition of FAW OS. 2525 genes are affected by OS. Some of these have previously been associated to the response to herbivore molecules. As exemple, the gene *OsLRR-RLK1* used in our study as marker for the herbivory (H list) displays a rapid up-regulated transcription after attack by the SSB or FAW OS deposit on leaves, but not to a mechanical wounding^16^. It has been suggested that *OsLRR-RLK1* is involved in the perception of a HAMP or an effector contained in FAW OS. This suggests that these 2525 genes respond specifically to HAMPs and/or effectors contained in OS.

As the GO enrichments of the three lists (D, H, DH) featured mostly terms associated to phytohormones, we wished then to understand if specific pathways were associated to either damage or herbivore response or both.

### Jasmonate pathway genes respond to both wounding and OS

**Figure 2:**
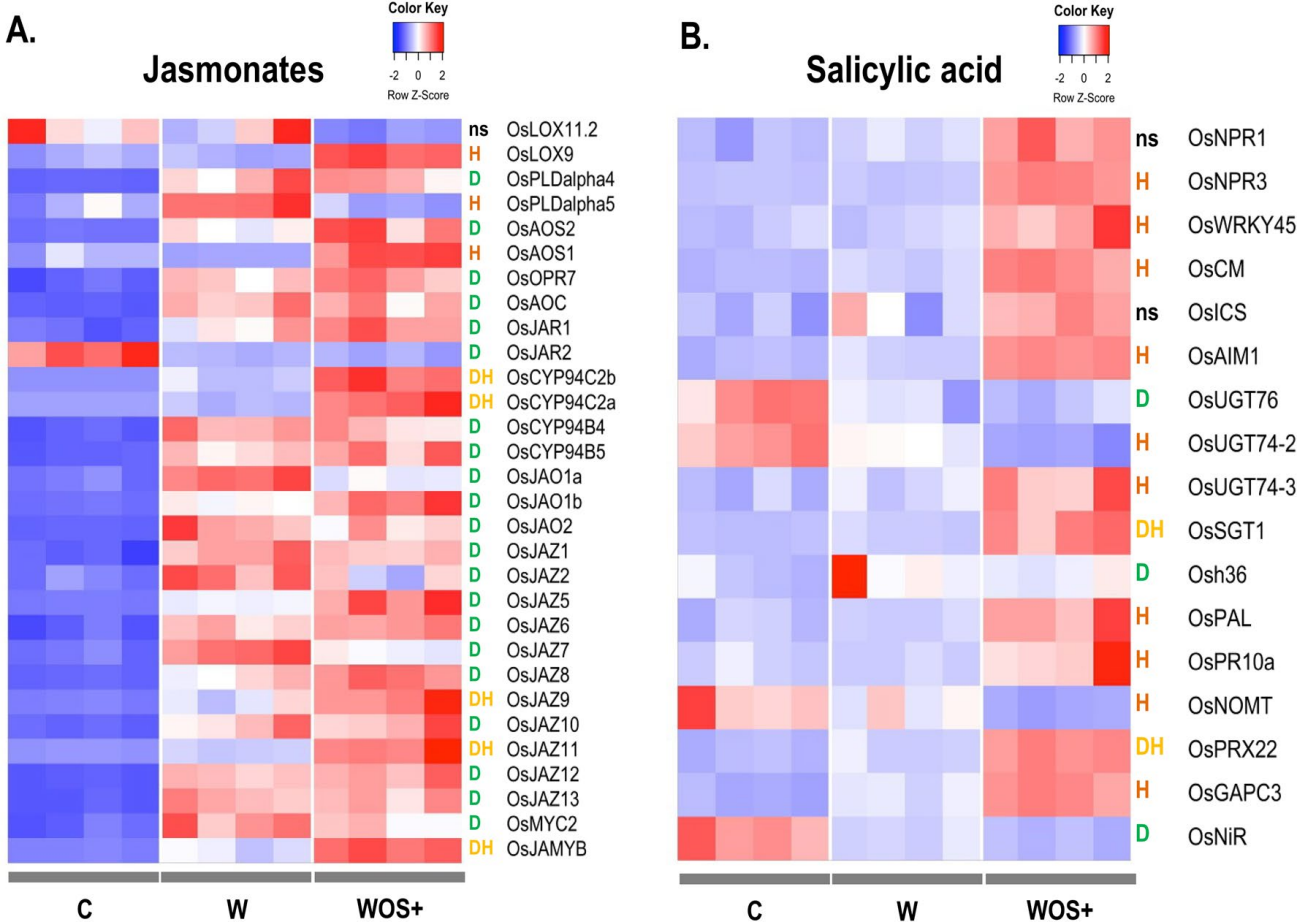
Transcriptional response of rice jasmonate (**A**) and salicylic acid (**B**) phytohormone-related pathways to FAW herbivory-mimick treatments. Heatmap of rice genes extracted from a literature-based list is shown. The letters indicate significative DEGs responding to damage only (D) and responding to the herbivore OS only (H). Genes affected by both effects are indicated as DH and genes whose transcriptional response is not significatively different in any condition are labeled with ns. The columns correspond to each replicate for the control (C), wounding (W) and wounding plus OS (WOS+) treatments, and rows for each gene manually annotated to belong to JA or SA pathways. The ID corresponding to each gene can be found in Supplementary Table 5. The color gradient represents z-scores of normalized counts for each gene.

Genes involved or related to phytohormone pathways are enriched in D, H and DH lists. To identify the key genes specifically responding to each component, we constituted a literature-based list of rice genes involved in JA, SA and other phytohormones pathways to vizualize their expression profile across our datasets. For the JA pathway, we annotated a list of 30 genes corresponding to JA biosynthesis, catabolism, perception and signaling (see Supplementary Table 5A for the full gene list and identifiers used in the subsequent figures). Most of the JA pathway genes are involved in wounding response as we observed the induction of 21 genes, either related to JA biosynthesis (*OsAOS2*, *OsOPR7*, *OsAOC*, *OsJAR1*, *OsPLDalpha4)*, JA catabolism *(OsCYP94B4*, *OsCYP94B5*, *OsJA01a*, *OsJAO1b*, *OsJAO2*) or genes encoding jasmonate-zim (JAZ) domain proteins involved in JA repression (*OsJAZ1, 2, 5, 6, 7, 8, 10, 12, 13*) (Fig. 2A). In rice, mechanical wounding usually induces the production of JA with a peak 30 min after wounding^19–21^, which correlates with our results of early JA-related transcriptionnal induction.

By contrast, the OS specifically induces the expression of only two JA biosynthesis related genes: *OsLOX9* and *OsAOS1* and it specifically represses the expression of one JA biosynthesis related gene (*OsPLDalpha5*) (Fig. 2A). Besides specific expression due to wounding or to OS, a co-modulation of expression is observed for five genes related to JA catabolism (*OsCYP94C2b, OsCYP94C2a*), JAZ repressors (*OsJAZ9*, *OsJAZ11*) and JA signaling (*OsJAMYB*) (Fig. 2A). Those genes are activated by wounding and their expression is enhanced by OS. Conversely, we observed that some genes induced by wounding showed their expression reduced in presence of OS, such as *OsJAZ7*, *OsPLDalpha5* or *OsJAO1a*. All these data indicate that OS can modify the JA response triggered by wounding in rice leaves.

While JA signalling is the major modulator in plant defense against herbivores^1^ and mechanical wounding results in a JA burst^19^, we showed that OS can also have a strong impact on the JA pathway. Our results therefore support previous observations with *S. mauritia* larvae. Deposition of their OS on wounded rice leaves induces a 1.5-fold accumulation of JA and JA-Ile two hours after treatment compared with wounded leaves^22^.

### Salicylic acid pathway is specifically induced by OS

Since SA was an enriched ontology in our DEGs, we constructed a list of SA-associated genes and analysed their expression patterns (Supplementary Table 5B and Fig. 2B). While wounding only induced *Osh36* and repressed *OsNiR* and *OsUGT76* transcription, OS has a strong impact on the number of modulated SA gene expression. It specifically induces eight SA markers *OsNPR3*, *OsWRKY45*, *OsAIM1*, *OsUGT74-3*, *OsPAL*, *OsPR10a*, *OsGAPC3* and *OsCM*. Two genes were under-expressed specifically by the OS treatement: *OsNOMT* and *OsUGT74-2* (Fig. 2B). A synergistic induction, although mainly driven by OS, is observed for two genes related to SA metabolism: *OsSGT1* and *OsPRX22* (Fig. 2B). In the literature, JA signaling is associated with wounding response to chewing insects^19,73^, while SA activation is responding to piercing-sucking insects^73^. We show here that SA signalling is also triggered by OS of chewing insects and to some extent by damage (Fig. 2B).

By contrast, we did not observe a clear association (Supplementary Fig. 2) between wounding or OS and modulation or co-modulation of specific genes from the AUX, BR, ET and CK hormones.

### Specific defense responses are activated by OS

**Figure 3:**
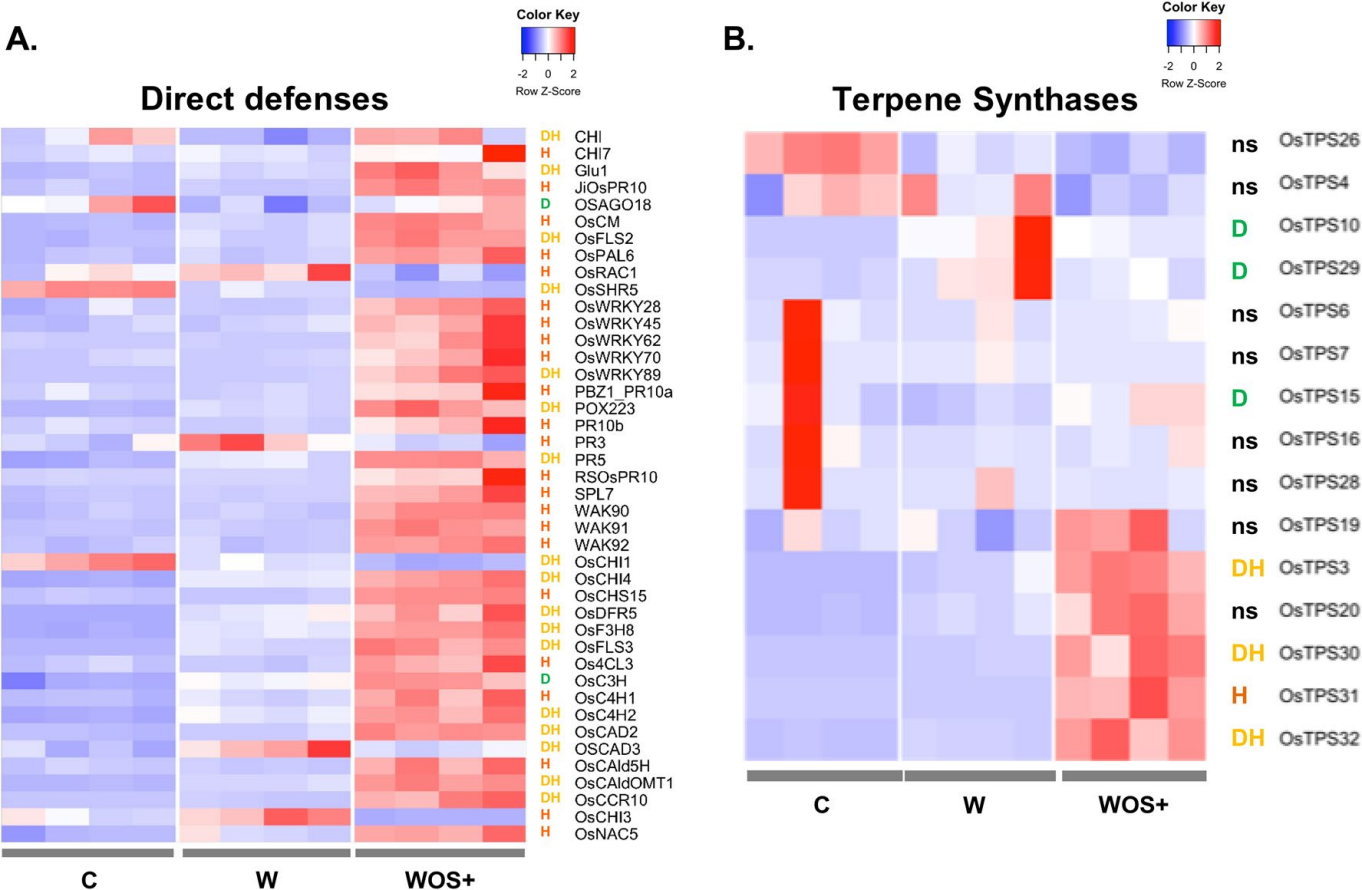
Transcriptional response of rice direct defenses (**A**) and terpene synthase (**B**) genes to FAW larvae herbivory-mimick treatments. Same legend as for Fig. 2.

Besides hormonal pathways, we investigated how the different components of herbivory affected expression of genes related to defense. We consolidated a non exhaustive list of known rice defense genes involved in PTI, ROS modulation or genes encoding pathogenesis related (PR) proteins^74^, and flavonoids biosynthesis enzymes (Supplementary Table 5D.E). Wounding induced the expression of eight ROS-encoding genes (*OsGSTF4*, *OsGSTU1, 6, 11, 24, 42* and *OsrbohA, H*) and repressed the expression of three ROS-encoding genes (*OsAPX9*, *OsGSTL2* and *OsGSTU10)* (Supplementary Fig. 3). It also induced two genes related to lignin metabolism *(OsCAD3* and *OsC3'H*) and repressed the PR gene *OsCHI* and the RNA silencing gene *OsAGO18* (Fig. 3A).

The OS, on the other hand, induced the flavonoid gene *OsCHS15*, four WRKY transcription factors (*OsWRKY70, 45, 28, 62*), six PR genes (*OsPR3*, *OsPR10b*, *OsPR10a*, *OsJiOSPR10*, *OsRSOsPR10* and *OsPAL6*), seven genes involved in basal immunity (*OsWAK90*, *91* and *92*, *OsCM*, *OsCHI, OSRAC1* and *OsCHI7)* and four genes involved in lignin metabolism (*OsNAC5*, *Os4CL3*, *OsCAld5H* and *OsC4H1*) (Fig. 3A). OS repressed the flavonoid gene *OsCHI3* and the lignin regulator *OsCAD3* (Fig. 3A). As expected, OS has a strong impact on SA-dependent PR genes and basal immunity-related genes. Remarkably, OS specifically induces the strong expression of four genes belonging to the PR10 gene family: *OsPR10a*, *OsPR10b*, *JiOsPR10* and *RSOsPR10*. *JiOsPR10* is known to respond to JA, SA and pathogenic infection and *RSOsPR10* is known to respond to biotic stress^75^. Those genes seem to have a specific role in the response to OS and can be considered as strong markers of the response to OS during herbivory.

In the DH list, several genes responding to wounding are further modulated by OS, including fifteen ROS genes (*OsGSTF1, 5*, *OsGSTU3, 5, 7, 31, 33, 35, 40, 48*, *OsCAtA*, *OsFSD1.1*, *OsGSTL1*, *OsGSTU13*) (Supplementary Fig. 3) and five genes involved in flavonoids synthesis (*OsDFR5, OsFLS3, OsF3H8, OsCHI4* and *OsCI1*) (Fig. 3A). While a certain level of co-modulation of direct defenses genes by wounding and OS exist, our results show that most direct defense genes are strongly activated by the deposition of the herbivore OS, suggesting that OS is a necessary cue for the plant to mount a specific response against herbivore.

Similarly, plants can use indirect defense systems such as volatile compounds to attract the natural enemies of the herbivore insect. We focused on a family of volatile compounds synthesized by terpene synthases (TPS) (Supplementary Table 5F). TPS produce terpenoids (monoterpenes, sesquiterpenes and diterpenes), molecules with phytoalexin and allelochemical functions^76^. Wounding specifically induced two TPS: *OsTSP10* and *OsTPS29* and repressed *OsTPS15* while OS modulated the expression of only *OsTPS31* (Fig. 3B). Three TPS (*OsTPS3*, *30* and *32*) were comodulated by wounding and OS (DH in Fig. 3B), but the heatmap shows that most of the activation resulted from the OS (Fig. 3B). *OsTPS3*, a monoterpene synthase, produces the (S)-linalool, which is known to be the most abundant volatile compound emitted by rice plants damaged by FAW larvae^76^. This compound is able to attract a generalist parasitic wasp (FAW natural enemy) *Cotesia marginiventris*^76^. In addition, *OsTPS3*, *OsTPS31* and *OsTPS29* are responsible for the majority of terpene production during FAW attack of rice^76^. In *Spodoptera exigua* (Hübner, 1808) OS, the FAC volicitin is known to stimulate the emission of volatile compounds^30,77^. It is conceivable that the induction of TPS genes by OS in our system is realized by the volicitin also present in FAW OS^78^.

Altogether, these results suggest that in addition to mechanical wounding, OS triggers an effective early defense responses of rice during herbivory by a chewing insect. As noted earlier, OS contain both HAMPs and effectors with different effects^78^ thus making it difficult to decipher the complex molecular interactions between plants and herbivores.

### Transcription factors modulated by wounding and OS

To globally estimate the transcriptional changes specifically regulated by D and H signals, we investigated the expression regulation of TFs. We first mapped all the DEGs to the PlantTFDB^79^ (http://planttfdb.gao-lab.org/) to retrieve a total of 297 D associated TFs encoding genes for which the expression of 236 TFs were over-expressed and 61 TFs were under-expressed (Supplementary Table 5G). In addition, OS application to wounded leaves affected specifically the expression of 209 TFs encoding genes, of which 118 genes were over-expressed and 91 genes were under-expressed (Supplementary Table 5H).

To further determine the BP affected by D and H signals, TF DEG lists were submitted to GO analysis (Supplementary Table 4G.H). Known damages responses genes, including genes involved in abiotic stress (“response to heat”, “response to deep water”, “cellular response to oxygen-containing compounds”), as well as many TFs involved in phytohormone action, such as AUX, BR, GA and ABA were enriched in wounded leaf. The transcriptional reprogramming of wounded leaf treated by OS is greatly different. For example, among over-expressed terms, different GO related to “response to herbivore”, “response to molecule of bacterial origin” and defense hormones (JA/SA) showed enrichment patterns. Interestingly, *OsMYC2*, which belongs to the bHLH family, known as a central TF in the core primary JA signaling pathway^80^ was found to be induced by wounding, but not affected by OS treatment. By contrast, *OsJAMYB* also well known to be involved in JA signalling and defense response^81^ is specifically induced by OS treatment suggesting that distinct JA-dependant regulators are driving transcriptional changes of rice plants under wounding and OS perception.

In addition to *OsJAMYB*, over-expressed TFs by herbivory OS, are also known to have a role in defense responses, notably the WRKY family. For example, *OsWRKY70* increases resistance against the SSB via the activation of JA-dependent defense pathways^18^. A study by Ye and co-workers^82^ shows that *OsWRKY70* is dependent on the *OsLRR-RLK1*-induced pathway via *OsMPK3*, upon attack by FAW. Furthermore, *OsWRKY70* is involved in the production of indole a volatile compound capable of attracting the natural enemies of this insect^82^. Other WRKY TFs over-expressed by OS are well known to be involved in defense response to pathogens such as *OsWRKY28*, *OsWRKY76* and *OsWRKY45* suggesting that OS containing microorganims could regulate plant immunity.

### Identification of the OS microbiota and dysbiosis

**Figure 4:**
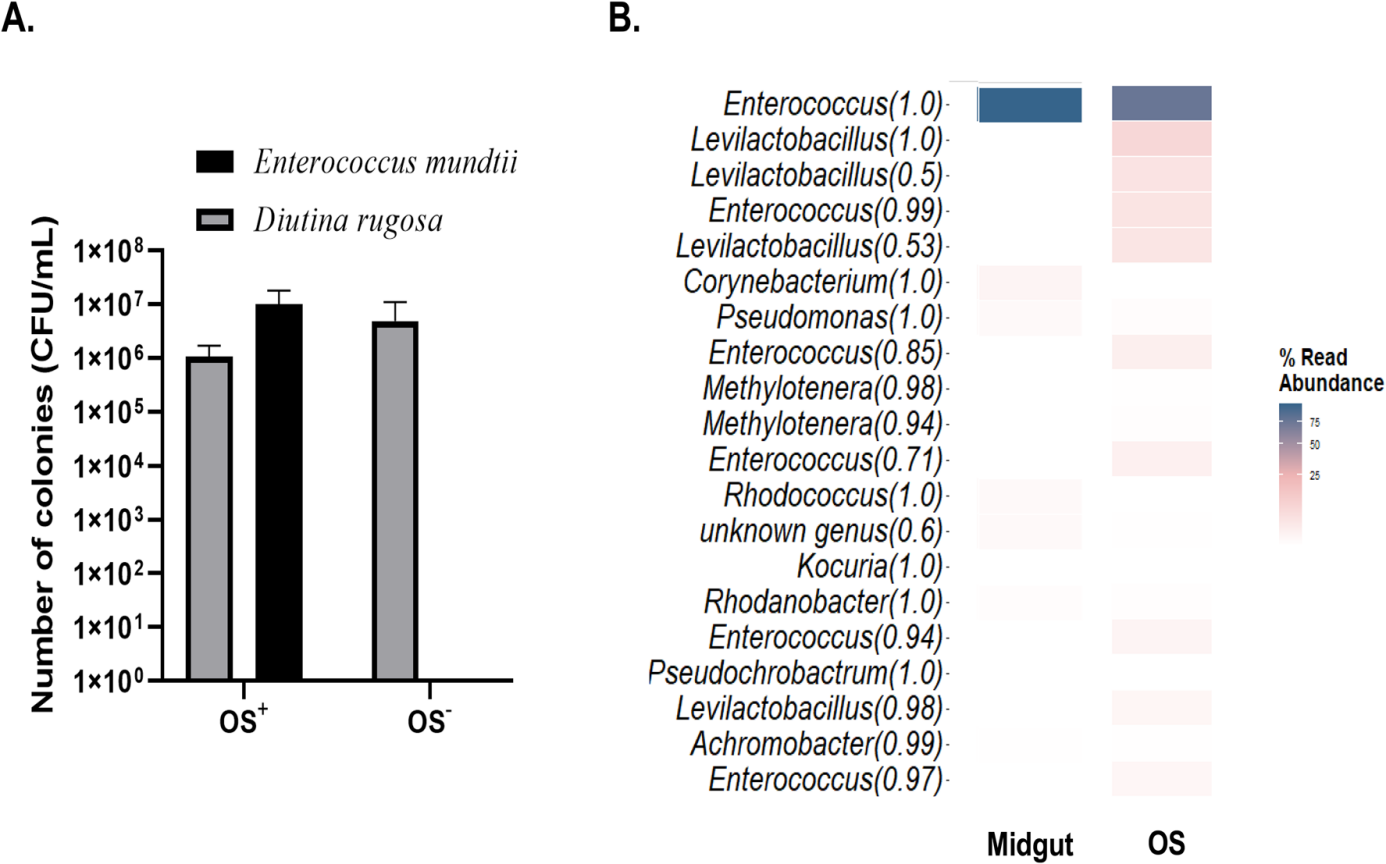
Bacterial community in the oral secretion (OS) of FAW larvae grown on artificial diet (Poitout). (**A**) Number of colonies of *Enterococcus mundtii* and *Diutina rugosa* in OS (OS +) and dysbiotic OS after antibiotic treatment of the diet (OS-). The two observed morphotypes were counted on nutrient agar medium and taxonomically identified after sequencing of the 16S rRNA gene. (**B**) Heatmap showing the bacterial microbial community composition of midgut and OS of FAW larvae. Each column is the mean of four (midgut) and three (OS) biological replicates. The 30 most abundant operational taxonomic units (OTUs) among OTUs with a minimal threshold of reads > 0.1% of the whole read are represented at the bacterial genus level. Their name is followed by a bootstrap confidence score, which gives a level of confidence to the assignment. The percentage of relative abundance is represented with a color gradient in log scale.

A further difficulty is the presence in the OS of a microbiota that could also be responsible for the modulation of early defense response during herbivory. Two studies have focused on the oral microbiota of FAW through culture approaches^11,83^, but neither has ever characterized the FAW oral microbiota by metabarcoding. To study the impact of bacteria contained in OS, we developed a procedure to modify the bacterial component of the FAW larvae OS by antibiotic treatement of the artificial diet (see Methods). To assess the impact of the treatment, we first used cultural approach. After plating of OS from untreated FAW, two microbial taxa repetitively grew on plates in similar abundance: the bacterium *Enterococcus mundtii* (Lactobacillales/Bacilli) and the yeast *Diutina rugosa* (Saccharomycetes/Sacharomycetales) (Fig. 4A). *E. mundtii*, a Gram^+^ bacterium of the phylum *Bacillota* (formely Firmicutes), is found in many *Lepidoptera* species at the intestinal level^84–87^. The yeast *D. rugosa* (formerly *Candida rugosa*) has been identified in larvae (whole body) of *Spodoptera litura* (Fabricius, 1775) collected from the field (castor crops) in India^88^. Yeasts are known to play a role in the supply of essential amino acids, toxin degradation and enzymatic digestion in many insect species including some belonging to the order *Lepidoptera*^89^*.*To check whether other non culturable species may also be present in OS, we performed metabarcoding analysis targeting the V3-V4 variable region of the 16S rRNA gene on OS samples. Midgut samples were used as a reference. We obtained 324,057 reads with an average of 32,406 reads per sample (Supplementary Fig. 4, Supplementary Table 6). The main taxa observed in midgut and OS from FAW belongs to the *Enterococcus* genus with the major part of the reads affiliated to this OTU, respectively 98.43% and 86.30% (Fig. 4B). The genus *Enterococcus* is very frequently associated to the FAW midgut and considered by some authors part of the core microbiota of FAW larvae even if the nature of this relationship was not understood yet^54^. There is a slight difference between the bacterial composition of OS and midgut. OS has minor OTUs affiliated with the genus *Levilactobacillus* (13.18% of reads), this bacterial genus is not present in FAW midgut. In conclusion, the *Enterococcus* genus is indeed the majority bacterial OTU in FAW OS. After antibiotic treatment of the FAW diet, the *E. mundtii* bacterium was not detected anymore in the OS and the yeast was not affected by the treatment (Fig. 4A). We used these dysbiotic OS to conduct rice leaves treatement (WOS− treatment) and assess the effect of the bacterial part of the OS on modulation of plant response.

### Transcriptomic modulation of rice leaves by OS microbiota

We performed a transcriptomic analysis as above and compared gene expression in leaves treated by OS+ or OS− after wounding. According to the PCA (Fig. 1A), WOS− treatment is different from C and W, but close to WOS+ treatment. When comparing WOS+ to WOS−, we obtained a list of 33 DEGs, four of which are over-expressed and 29 are under-expressed (Supplementary Table 5I). We verified this effect by quantitative RT-PCR on three genes from this list: *OsBURP02*, *OsLTPL145* and *OsPhiP* (Supplementary Fig. 1D). The comparison histograms between normalized counts and ΔΔCt are different, as the expression level of the transcripts in qPCR was very low and difficult to detect, as the genes are poorly expressed (Supplementary Fig. 1D). No GO enrichment could be observed in this list due to the small size of the list.

**Figure 5:**
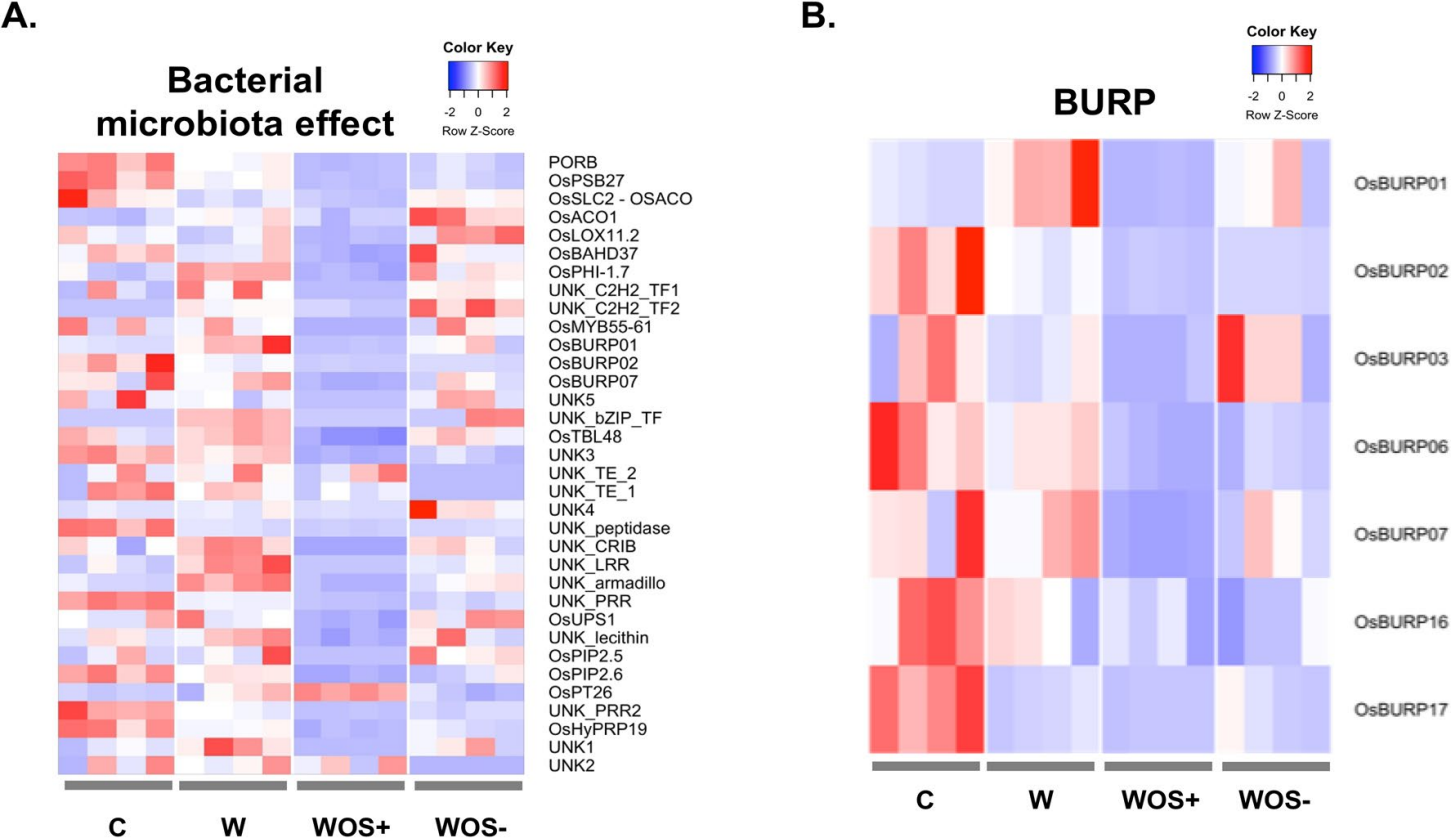
Differential expressed genes (DEGs) observed between the wounding plus oral secretion treatment (WOS+) and the wounding plus dysbiotic oral secretion treatment (WOS−). (**A**) Heatmap of the thirty-three DEGs associated with a bacterial microbiota effect is shown. (**B**) Specific heatmap with seven *OsBURP* genes expressed in leaves in our study, among which *OsBURP01, OsBURP02 and OsBURP07*, three DEGs associated with a bacterial microbiota effect. Same legend as in Fig. 2.

Since OS are mainly composed of *Enterococcus* and since antibiotic treatment substantially removes the *E. mundtii* species from OS, we can assume that at least this bacterial species participates to the transcriptional repression of the 29 DEGs in WOS+ condition. Interestingly, among the under-expressed genes were a lipoxygenase involved in JA biosynthesis (*OsLOX2.1*) (Fig. 5A), and some genes involved in the metabolism of other phytohormones: two ACC (amino cyclo propane carboxylate) oxidase (*OsACO*, *OsACO1*) involved in ET biosynthesis, a phosphate-induced protein that responds to ABA, BR and ET (*OsPHI-1.7*) and a gene involved in SA production (*OsSLC2*) (Fig. 5A). While phytohormone synthesis seems to be negatively affected by microbiota, we did not observe a downstream effect on the production of direct defenses, except OsBAHD37 an acyltransferase involved in the acylation of secondary metabolites, nor indirect defenses, with no effect on TPS 2 h after treatment. This suggests that the early response in rice is mainly driven by the insect component of the OS rather than by the OS microbiota. Interestingly, the list of 33 DEGs contains three under-expressed genes belonging to the BURP domain containing protein family, *OsBURP01*, *OsBURP02* and *OsBURP07*, a gene family represented by 17 members in rice^90^ (Fig. 5A and Supplementary Table 5 J), eight of which are known to be expressed in leaves (*OsBURP01, 02, 03, 06, 07, 08, 16, 17*) (Fig. 5B). In our dataset, while *OsBURP01*, *OsBURP02* and *OsBURP07* are significatively repressed by OS microbiota, we could observe that all *OsBURP* are repressed in WOS+ treatment. Wounding alone seems to induce *OsBURP01* but represses *OsBURP17*. No expression is detected in our dataset for *OsBURP08*. *OsBURP* genes are known to play a role in plant vegetative and reproductive development and in tolerance to abiotic stresses (drought, salt stress, aluminum and cold) and respond to ABA^90^. In addition, *OsBURP02* and *OsBURP07* are induced 24 h after infestation by rice leafolder (*Cnaphalocrocis medinalis*, Guenée, 1854, chewing insect) and brown planthopper (*Nilparvata lugens*, Stahl, 1854, piercing-sucking insect)^33^. In rice, this induction of *OsBURP* might be prevented by the OS microbiota. The functional relevance of *OsBURP* in response to herbivory remains to be investigated.

## Conclusion

When faced with an insect herbivore attack, the plant must identify and integrate a variety of signals to specifically regulate a repertoire of defenses. In this study, we described the early transcriptomic response of rice to the different components of herbivory by FAW, a chewing insect: mechanical wounding, OS and oral microbiota. We identified hundreds of genes in rice differentially regulated by mechanical wounding or the presence of insect OS. The application of OS on the wound drastically modifies the expression of genes related to direct defense responses (flavonoids, PR genes, basal immunity, lignin). The DAMPs, HAMPs and effectors at the origin of these responses remain to be identified. We also correlated the perturbation of the the oral microbiota with the attenuation of the expression of some key genes involved in herbivory response, including JA biosynthesis as well as genes whose function remains unknown in this context, such as the BURP family genes. Further studies must be conducted to determine whether the modulation of those key genes could affect the performance of FAW larvae feeding on rice and to understand better the efficiency of rice immunity against herbivores.

To understand the multitrophic interaction between FAW, its oral microbiota and rice, it would be relevant to focus further studies on JA, the central hormone of the response to herbivory. In particular, by studying the ability of the FAW bacterial microbiota to suppress the JA pathway in rice, as has been shown in response to *C. suppresalis* attack^25^, as well as to study the impact of JA on FAW fitness.

### Supplementary Information

Supplementary Information 1.

Supplementary Information 2.

Supplementary Information 3.

Supplementary Information 4.

Supplementary Information 5.

## Data Availability

Raw fastq files can be found on ArrayExpress https://www.ebi.ac.uk/biostudies/arrayexpress/studies/E-MTAB-13227?key=ecc997c3-d8aa-476d-85a5-601642df3c19.
