# Supplementary material for: Early transcriptomic responses of rice leaves to herbivory by Spodoptera frugiperda
Source: Sci Rep. 2024 Feb 3;14:2836. doi: 10.1038/s41598-024-53348-x (PMC10838271; doi:10.1038/s41598-024-53348-x)
Supplement: Supplementary file 1 — Supplementary Information 1. [file 41598_2024_53348_MOESM1_ESM.pptx]

## Slide 1
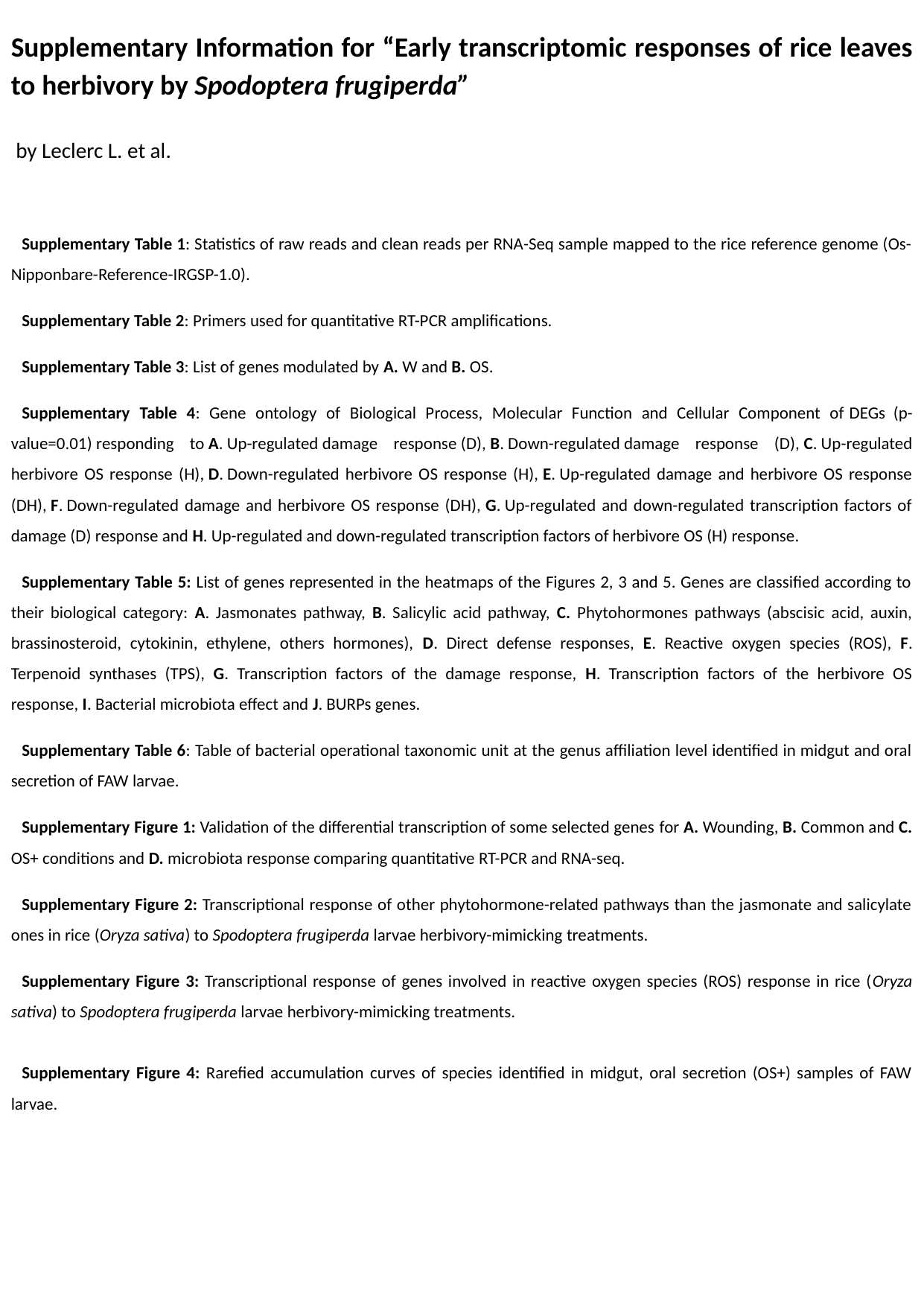

Supplementary Information for “Early transcriptomic responses of rice leaves to herbivory by Spodoptera frugiperda”
 by Leclerc L. et al.
Supplementary Table 1: Statistics of raw reads and clean reads per RNA-Seq sample mapped to the rice reference genome (Os-Nipponbare-Reference-IRGSP-1.0).
Supplementary Table 2: Primers used for quantitative RT-PCR amplifications.
Supplementary Table 3: List of genes modulated by A. W and B. OS.
Supplementary Table 4: Gene ontology of Biological Process, Molecular Function and Cellular Component of DEGs  (p-value=0.01) responding to A. Up-regulated damage response (D), B. Down-regulated damage response (D), C. Up-regulated herbivore OS response (H), D. Down-regulated herbivore OS response (H), E. Up-regulated damage and herbivore OS response (DH), F. Down-regulated damage and herbivore OS response (DH), G. Up-regulated and down-regulated transcription factors of damage (D) response and H. Up-regulated and down-regulated transcription factors of herbivore OS (H) response.
Supplementary Table 5: List of genes represented in the heatmaps of the Figures 2, 3 and 5. Genes are classified according to their biological category: A. Jasmonates pathway, B. Salicylic acid pathway, C. Phytohormones pathways (abscisic acid, auxin, brassinosteroid, cytokinin, ethylene, others hormones), D. Direct defense responses, E. Reactive oxygen species (ROS), F. Terpenoid synthases (TPS), G. Transcription factors of the damage response, H. Transcription factors of the herbivore OS response, I. Bacterial microbiota effect and J. BURPs genes.
Supplementary Table 6: Table of bacterial operational taxonomic unit at the genus affiliation level identified in midgut and oral secretion of FAW larvae.
Supplementary Figure 1: Validation of the differential transcription of some selected genes for A. Wounding, B. Common and C. OS+ conditions and D. microbiota response comparing quantitative RT-PCR and RNA-seq.
Supplementary Figure 2: Transcriptional response of other phytohormone-related pathways than the jasmonate and salicylate ones in rice (Oryza sativa) to Spodoptera frugiperda larvae herbivory-mimicking treatments.
Supplementary Figure 3: Transcriptional response of genes involved in reactive oxygen species (ROS) response in rice (Oryza sativa) to Spodoptera frugiperda larvae herbivory-mimicking treatments.
Supplementary Figure 4: Rarefied accumulation curves of species identified in midgut, oral secretion (OS+) samples of FAW larvae.

## Slide 2
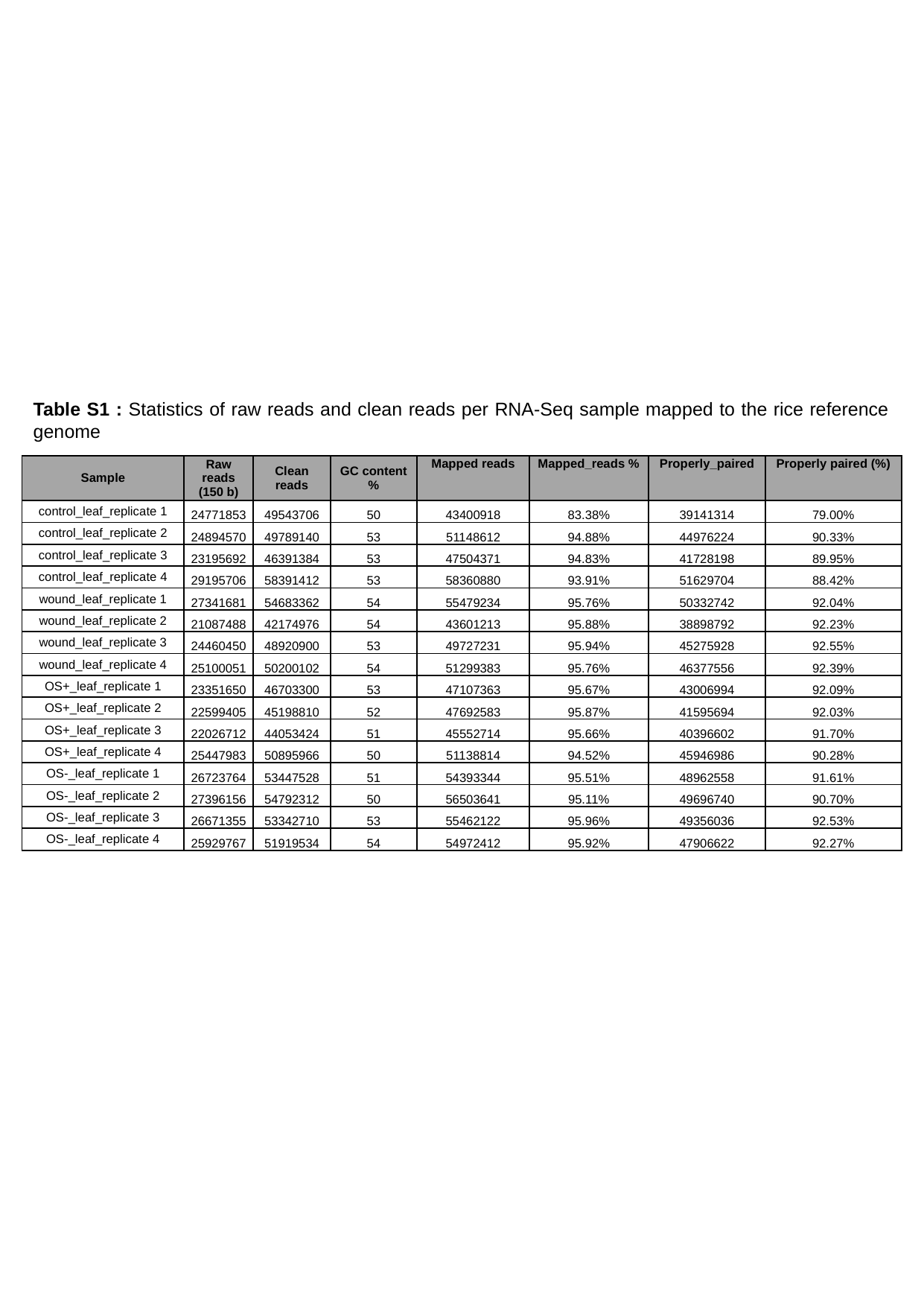

Table S1 : Statistics of raw reads and clean reads per RNA-Seq sample mapped to the rice reference genome
| Sample | Raw reads (150 b) | Clean reads | GC content % | Mapped reads | Mapped\_reads % | Properly\_paired | Properly paired (%) |
| --- | --- | --- | --- | --- | --- | --- | --- |
| control\_leaf\_replicate 1 | 24771853 | 49543706 | 50 | 43400918 | 83.38% | 39141314 | 79.00% |
| control\_leaf\_replicate 2 | 24894570 | 49789140 | 53 | 51148612 | 94.88% | 44976224 | 90.33% |
| control\_leaf\_replicate 3 | 23195692 | 46391384 | 53 | 47504371 | 94.83% | 41728198 | 89.95% |
| control\_leaf\_replicate 4 | 29195706 | 58391412 | 53 | 58360880 | 93.91% | 51629704 | 88.42% |
| wound\_leaf\_replicate 1 | 27341681 | 54683362 | 54 | 55479234 | 95.76% | 50332742 | 92.04% |
| wound\_leaf\_replicate 2 | 21087488 | 42174976 | 54 | 43601213 | 95.88% | 38898792 | 92.23% |
| wound\_leaf\_replicate 3 | 24460450 | 48920900 | 53 | 49727231 | 95.94% | 45275928 | 92.55% |
| wound\_leaf\_replicate 4 | 25100051 | 50200102 | 54 | 51299383 | 95.76% | 46377556 | 92.39% |
| OS+\_leaf\_replicate 1 | 23351650 | 46703300 | 53 | 47107363 | 95.67% | 43006994 | 92.09% |
| OS+\_leaf\_replicate 2 | 22599405 | 45198810 | 52 | 47692583 | 95.87% | 41595694 | 92.03% |
| OS+\_leaf\_replicate 3 | 22026712 | 44053424 | 51 | 45552714 | 95.66% | 40396602 | 91.70% |
| OS+\_leaf\_replicate 4 | 25447983 | 50895966 | 50 | 51138814 | 94.52% | 45946986 | 90.28% |
| OS-\_leaf\_replicate 1 | 26723764 | 53447528 | 51 | 54393344 | 95.51% | 48962558 | 91.61% |
| OS-\_leaf\_replicate 2 | 27396156 | 54792312 | 50 | 56503641 | 95.11% | 49696740 | 90.70% |
| OS-\_leaf\_replicate 3 | 26671355 | 53342710 | 53 | 55462122 | 95.96% | 49356036 | 92.53% |
| OS-\_leaf\_replicate 4 | 25929767 | 51919534 | 54 | 54972412 | 95.92% | 47906622 | 92.27% |

## Slide 3
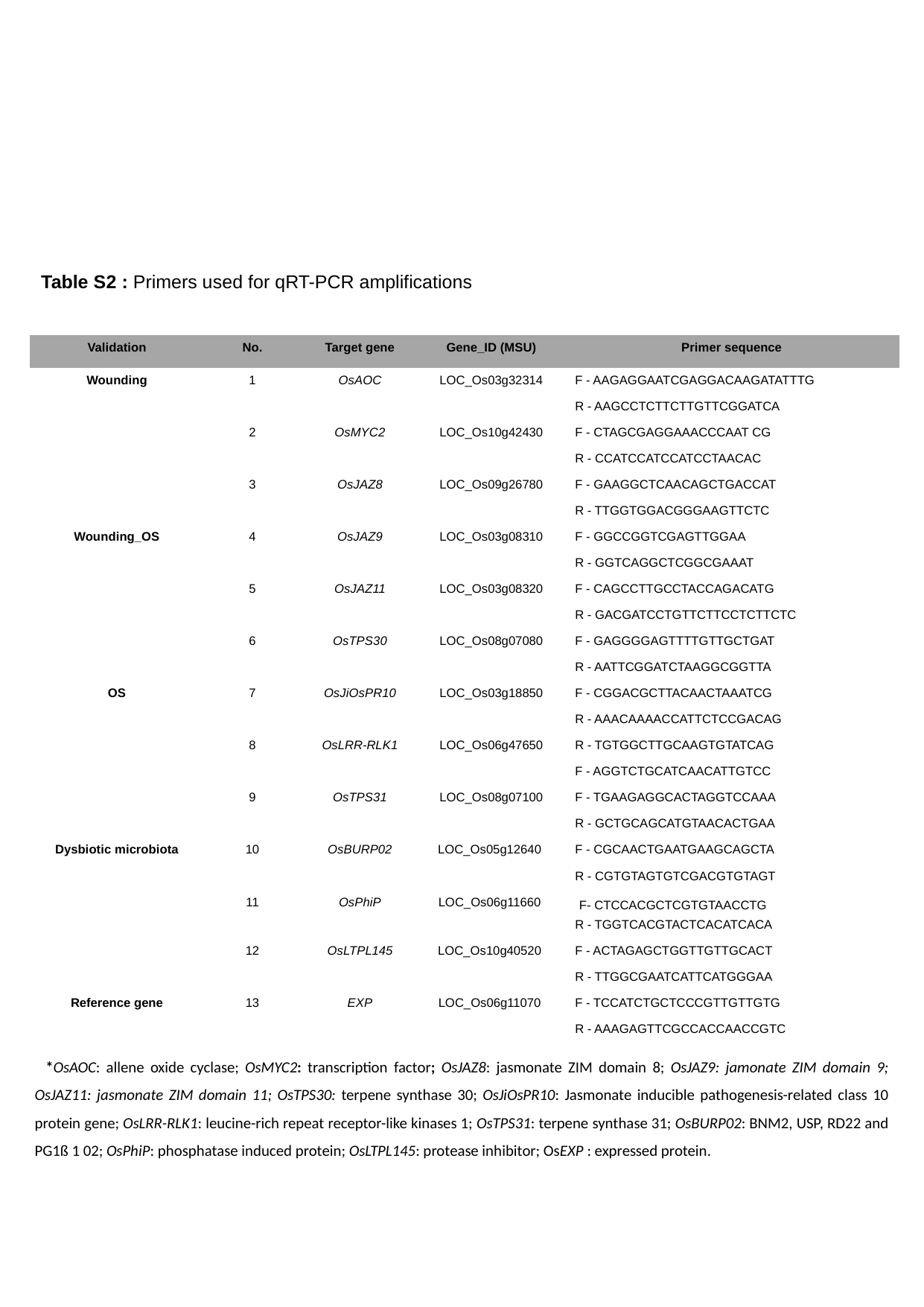

Table S2 : Primers used for qRT-PCR amplifications
| Validation | No. | Target gene | Gene\_ID (MSU) | Primer sequence |
| --- | --- | --- | --- | --- |
| Wounding | 1 | OsAOC | LOC\_Os03g32314 | F - AAGAGGAATCGAGGACAAGATATTTG |
| | | | | R - AAGCCTCTTCTTGTTCGGATCA |
| | 2 | OsMYC2 | LOC\_Os10g42430 | F - CTAGCGAGGAAACCCAAT CG |
| | | | | R - CCATCCATCCATCCTAACAC |
| | 3 | OsJAZ8 | LOC\_Os09g26780 | F - GAAGGCTCAACAGCTGACCAT |
| | | | | R - TTGGTGGACGGGAAGTTCTC |
| Wounding\_OS | 4 | OsJAZ9 | LOC\_Os03g08310 | F - GGCCGGTCGAGTTGGAA |
| | | | | R - GGTCAGGCTCGGCGAAAT |
| | 5 | OsJAZ11 | LOC\_Os03g08320 | F - CAGCCTTGCCTACCAGACATG |
| | | | | R - GACGATCCTGTTCTTCCTCTTCTC |
| | 6 | OsTPS30 | LOC\_Os08g07080 | F - GAGGGGAGTTTTGTTGCTGAT |
| | | | | R - AATTCGGATCTAAGGCGGTTA |
| OS | 7 | OsJiOsPR10 | LOC\_Os03g18850 | F - CGGACGCTTACAACTAAATCG |
| | | | | R - AAACAAAACCATTCTCCGACAG |
| | 8 | OsLRR-RLK1 | LOC\_Os06g47650 | R - TGTGGCTTGCAAGTGTATCAG |
| | | | | F - AGGTCTGCATCAACATTGTCC |
| | 9 | OsTPS31 | LOC\_Os08g07100 | F - TGAAGAGGCACTAGGTCCAAA |
| | | | | R - GCTGCAGCATGTAACACTGAA |
| Dysbiotic microbiota | 10 | OsBURP02 | LOC\_Os05g12640 | F - CGCAACTGAATGAAGCAGCTA |
| | | | | R - CGTGTAGTGTCGACGTGTAGT |
| | 11 | OsPhiP | LOC\_Os06g11660 | F- CTCCACGCTCGTGTAACCTG |
| | | | | R - TGGTCACGTACTCACATCACA |
| | 12 | OsLTPL145 | LOC\_Os10g40520 | F - ACTAGAGCTGGTTGTTGCACT |
| | | | | R - TTGGCGAATCATTCATGGGAA |
| Reference gene | 13 | EXP | LOC\_Os06g11070 | F - TCCATCTGCTCCCGTTGTTGTG |
| | | | | R - AAAGAGTTCGCCACCAACCGTC |
*OsAOC: allene oxide cyclase; OsMYC2: transcription factor; OsJAZ8: jasmonate ZIM domain 8; OsJAZ9: jamonate ZIM domain 9; OsJAZ11: jasmonate ZIM domain 11; OsTPS30: terpene synthase 30; OsJiOsPR10: Jasmonate inducible pathogenesis-related class 10 protein gene; OsLRR-RLK1: leucine-rich repeat receptor-like kinases 1; OsTPS31: terpene synthase 31; OsBURP02: BNM2, USP, RD22 and PG1ß 1 02; OsPhiP: phosphatase induced protein; OsLTPL145: protease inhibitor; OsEXP : expressed protein.

## Slide 4
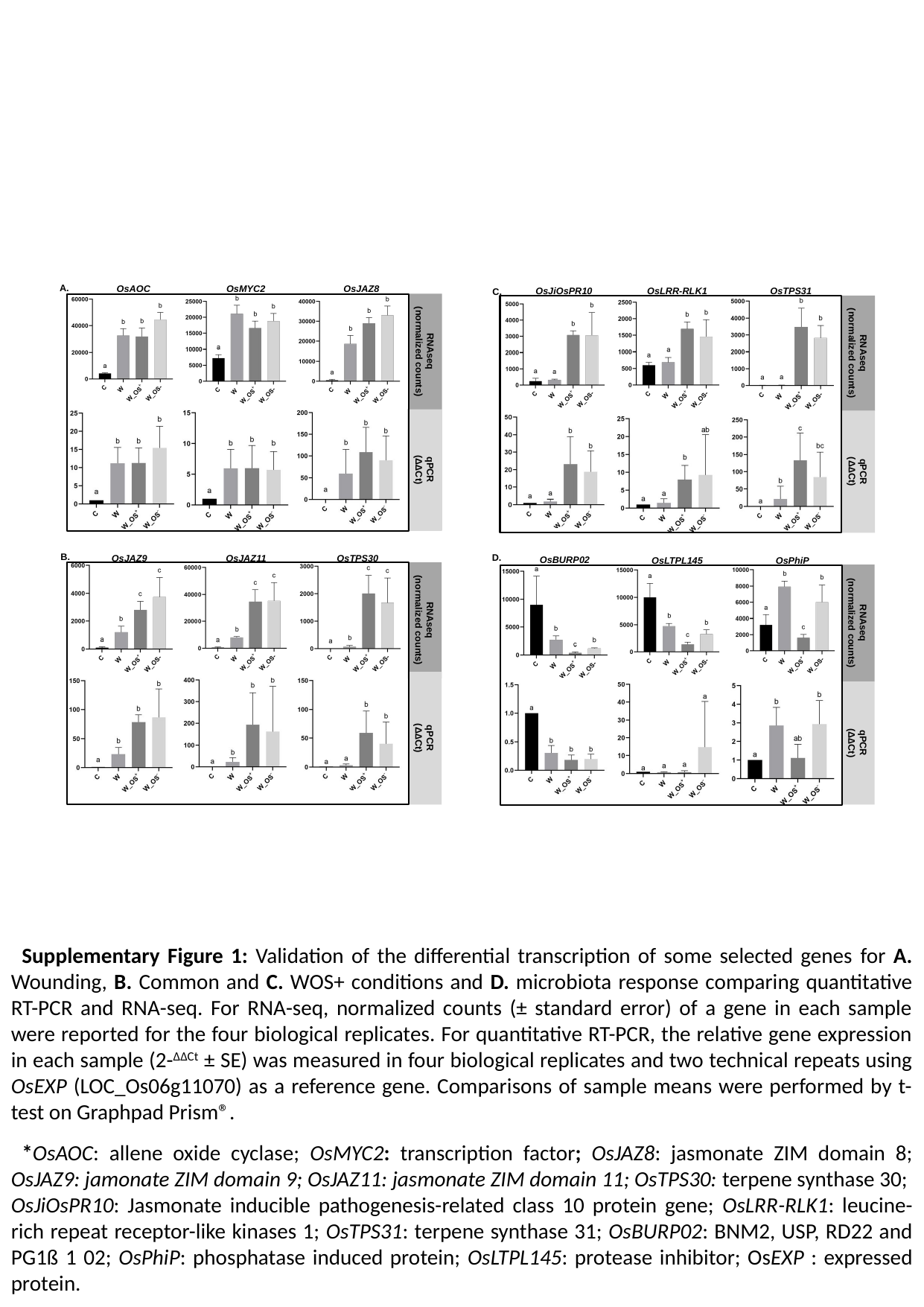

A.
OsAOC
OsJAZ8
OsMYC2
OsLRR-RLK1
OsJiOsPR10
OsTPS31
C.
RNAseq
(normalized counts)
RNAseq
(normalized counts)
qPCR
(Δ∆Ct)
qPCR
(Δ∆Ct)
B.
D.
OsTPS30
OsJAZ9
OsJAZ11
OsBURP02
OsPhiP
OsLTPL145
RNAseq
(normalized counts)
RNAseq
(normalized counts)
qPCR
(Δ∆Ct)
qPCR
(Δ∆Ct)
Supplementary Figure 1: Validation of the differential transcription of some selected genes for A. Wounding, B. Common and C. WOS+ conditions and D. microbiota response comparing quantitative RT-PCR and RNA-seq. For RNA-seq, normalized counts (± standard error) of a gene in each sample were reported for the four biological replicates. For quantitative RT-PCR, the relative gene expression in each sample (2-Δ∆Ct ± SE) was measured in four biological replicates and two technical repeats using OsEXP (LOC_Os06g11070) as a reference gene. Comparisons of sample means were performed by t-test on Graphpad Prism®.
*OsAOC: allene oxide cyclase; OsMYC2: transcription factor; OsJAZ8: jasmonate ZIM domain 8; OsJAZ9: jamonate ZIM domain 9; OsJAZ11: jasmonate ZIM domain 11; OsTPS30: terpene synthase 30; OsJiOsPR10: Jasmonate inducible pathogenesis-related class 10 protein gene; OsLRR-RLK1: leucine-rich repeat receptor-like kinases 1; OsTPS31: terpene synthase 31; OsBURP02: BNM2, USP, RD22 and PG1ß 1 02; OsPhiP: phosphatase induced protein; OsLTPL145: protease inhibitor; OsEXP : expressed protein.

## Slide 5
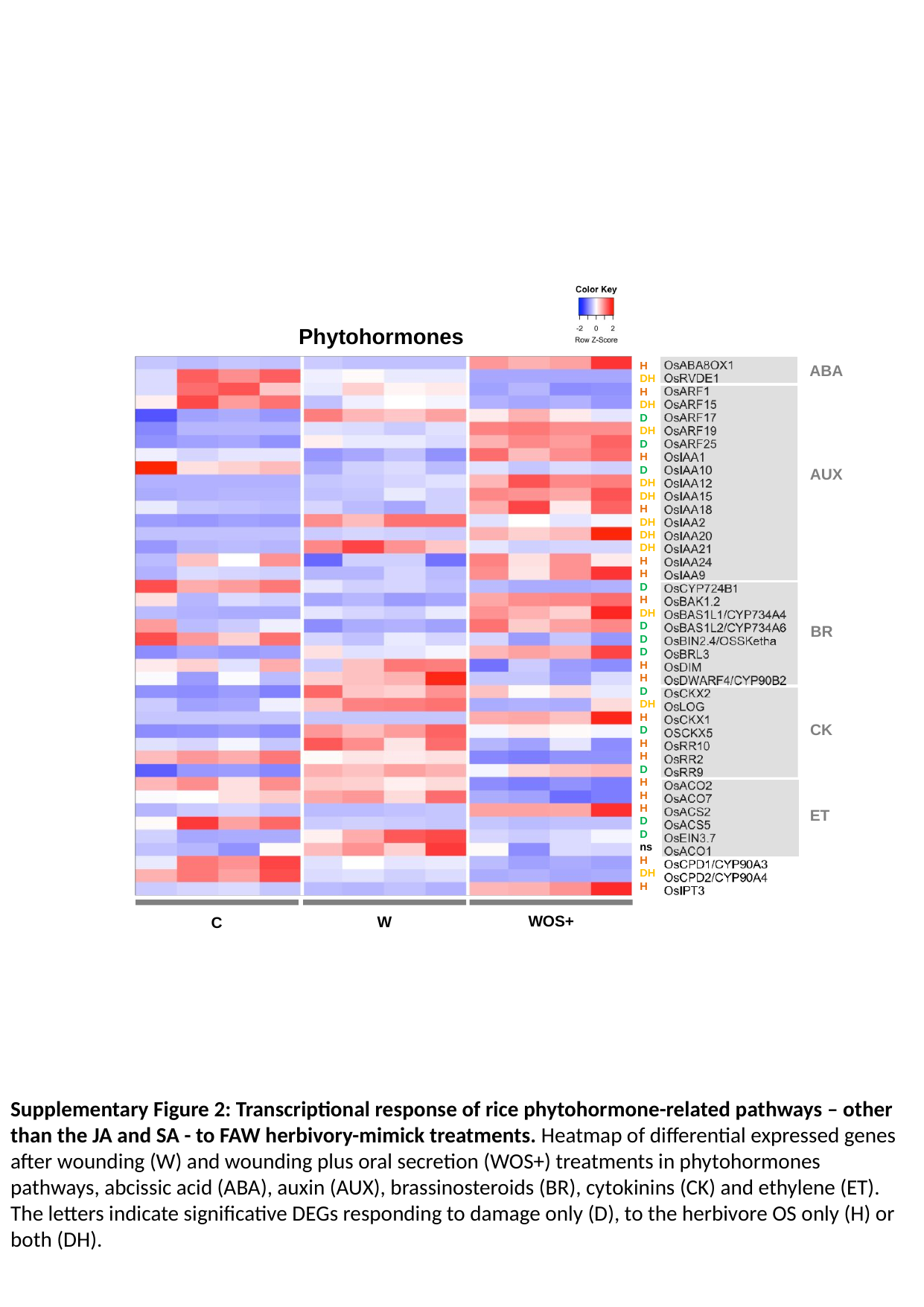

Phytohormones
H
DH
H
DH
D
DH
D
H
D
DH
DH
H
DH
DH
DH
H
H
D
H
DH
D
D
D
H
H
D
DH
H
D
H
H
D
H
H
H
D
D
ns
H
DH
H
ABA
AUX
BR
CK
ET
WOS+
W
C
Supplementary Figure 2: Transcriptional response of rice phytohormone-related pathways – other than the JA and SA - to FAW herbivory-mimick treatments. Heatmap of differential expressed genes after wounding (W) and wounding plus oral secretion (WOS+) treatments in phytohormones pathways, abcissic acid (ABA), auxin (AUX), brassinosteroids (BR), cytokinins (CK) and ethylene (ET). The letters indicate significative DEGs responding to damage only (D), to the herbivore OS only (H) or both (DH).

## Slide 6
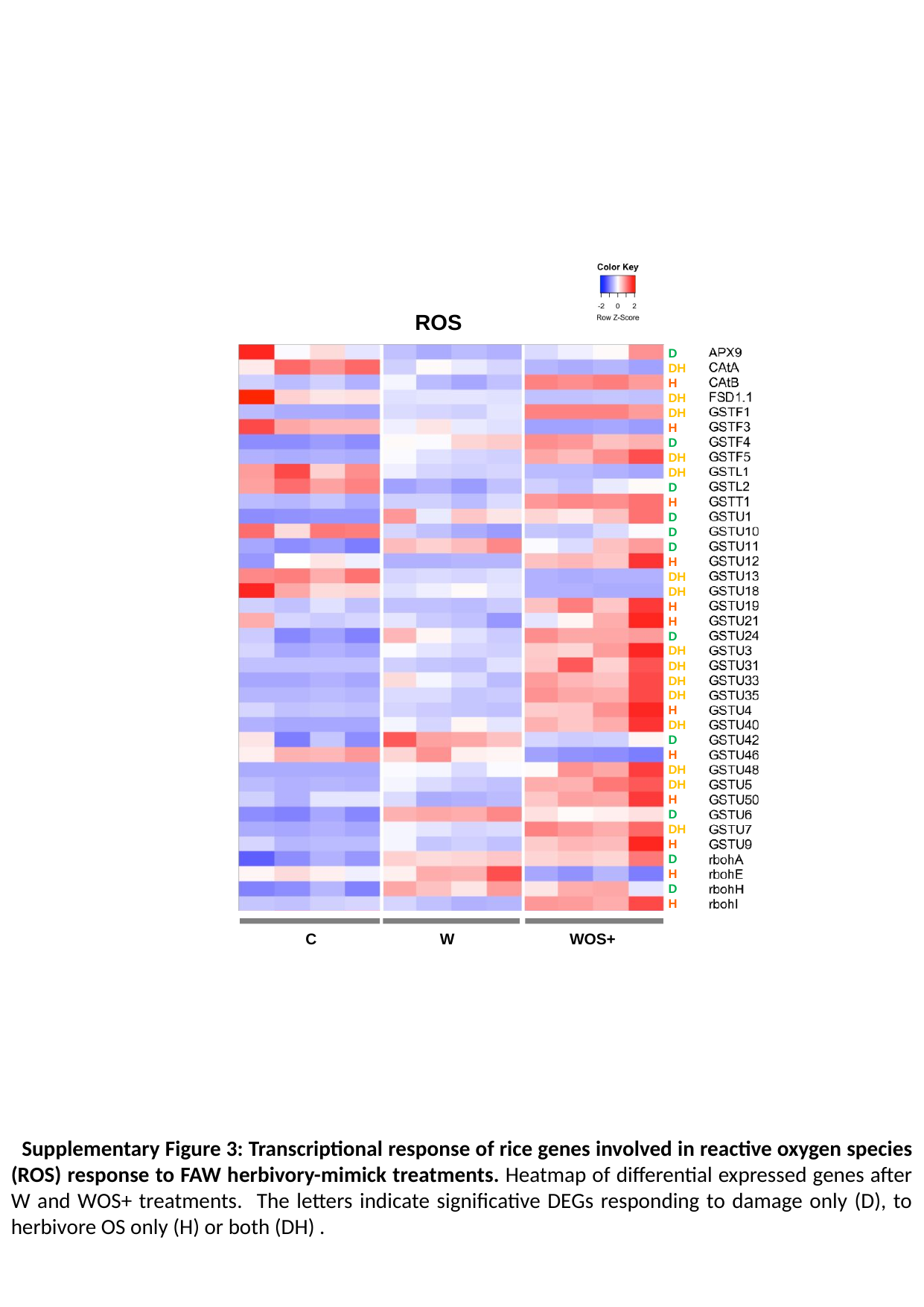

ROS
D
DH
H
DH
DH
H
D
DH
DH
D
H
D
D
D
H
DH
DH
H
H
D
DH
DH
DH
DH
H
DH
D
H
DH
DH
H
D
DH
H
D
H
D
H
C
W
WOS+
Supplementary Figure 3: Transcriptional response of rice genes involved in reactive oxygen species (ROS) response to FAW herbivory-mimick treatments. Heatmap of differential expressed genes after W and WOS+ treatments. The letters indicate significative DEGs responding to damage only (D), to herbivore OS only (H) or both (DH) .

## Slide 7
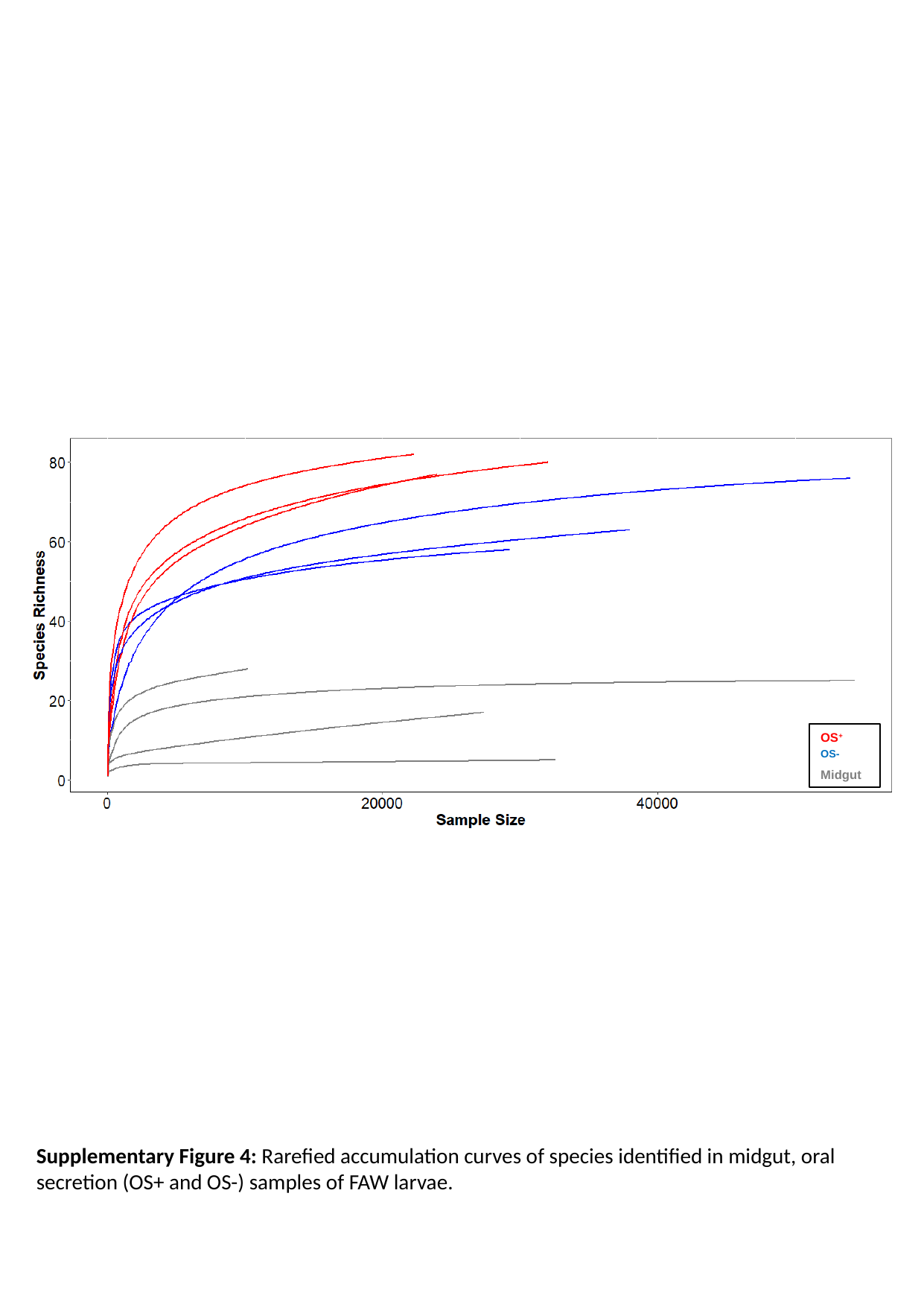

OS+
OS-
Midgut
Supplementary Figure 4: Rarefied accumulation curves of species identified in midgut, oral secretion (OS+ and OS-) samples of FAW larvae.
